# Principal Component Analysis of Oxford Cognitive Screen in Patients With Stroke

**DOI:** 10.3389/fneur.2022.779679

**Published:** 2022-05-27

**Authors:** Marco Iosa, Nele Demeyere, Laura Abbruzzese, Pierluigi Zoccolotti, Mauro Mancuso

**Affiliations:** ^1^Department of Psychology, Sapienza University of Rome, Rome, Italy; ^2^IRCCS Fondazione Santa Lucia, Rome, Italy; ^3^Department of Experimental Psychology, University of Oxford, Oxford, United Kingdom; ^4^Tuscany Rehabilitation Clinic, Arezzo, Italy; ^5^Physical and Rehabilitative Medicine Unit, NHS-USL Tuscany South-Est, Grosseto, Italy

**Keywords:** cognition, stroke, rehabilitation, psychometrics, assessment

## Abstract

Cognitive deficits occur in most patients with stroke and are the important predictors of adverse long-term outcome. Early identification is fundamental to plan the most appropriate care, including rehabilitation and discharge decisions. The Oxford Cognitive Screen (OCS) is a simple, valid, and reliable tool for the assessment of cognitive deficits in patients with stroke. It contains 10 subtests, providing 14 scores referring to 5 theoretically derived cognitive domains: attention, language, number, praxis, and memory. However, an empirical verification of the domain composition of the OCS subtests in stroke data is still lacking in the literature. A principal component analysis (PCA) was performed on 1,973 patients with stroke who were enrolled in OCS studies in the UK and in Italy. A number of six main components were identified relating to the domains of language and arithmetic, memory, visuomotor ability, orientation, spatial exploration, and executive functions. Bootstrapped split-half reliability analysis on patients and comparison between patients and 498 healthy participants, as that between patients with left and right hemisphere damage, confirmed the results obtained by the principal component analysis. A clarification about the contribution of each score to the theoretical original domains and to the components identified by the PCA is provided with the aim to foster the usability of OCS for both clinicians and researchers.

## Introduction

Cognitive deficits occur in 50–78% of patients with stroke (1), and their early identification is fundamental to plan the most appropriate neurorehabilitation program (2). The Oxford Cognitive Screen (OCS) is a screening tool providing a “snapshot” of the patient's cognitive profile helpful for designing the rehabilitation program according to the patient's needs (3). The OCS entails 10 subtests: picture naming, semantics, orientation, visual field, sentence reading, number writing and calculation, broken hearts, imitation, recall and recognition, and trails. It is easy to administer and score, takes a relatively short time, can be delivered at the bedside, and can be administered in the acute phase (3, 4).

The Oxford Cognitive Screen was initially tested on 140 neurologically healthy English participants and 208 acute patients with stroke demonstrating its reliability, convergent and divergent validity, and sensitivity in differentiating between patients with right vs. left brain damage (4). In a successive study (5) on 200 patients with stroke, the OCS was shown to be more sensitive than the Montreal Cognitive Assessment (MoCA) in highlighting cognitive impairments in this type of patients. In addition, OCS was found to be more inclusive for participants with aphasia and not dominated (as MoCA) by left hemisphere impairments, instead of giving differentiated profiles across the contrasting domains. Similar results on patients with stroke were obtained by the comparison of OCS with the Mini-Mental State Examination (6). Overall, the OCS detects important cognitive deficits after stroke not assessed in standard cognitive screening developed for dementia, it is inclusive for patients with aphasia and neglect, and it is less confounded by co-occurring difficulties in these domains.

The OCS has been validated and standardized in many other languages, including Italian (4), Spanish (7), Brazilian Portuguese (8), Chinese (9), Dutch (10), Russian (11), and Danish (12).

**Figure 1:**
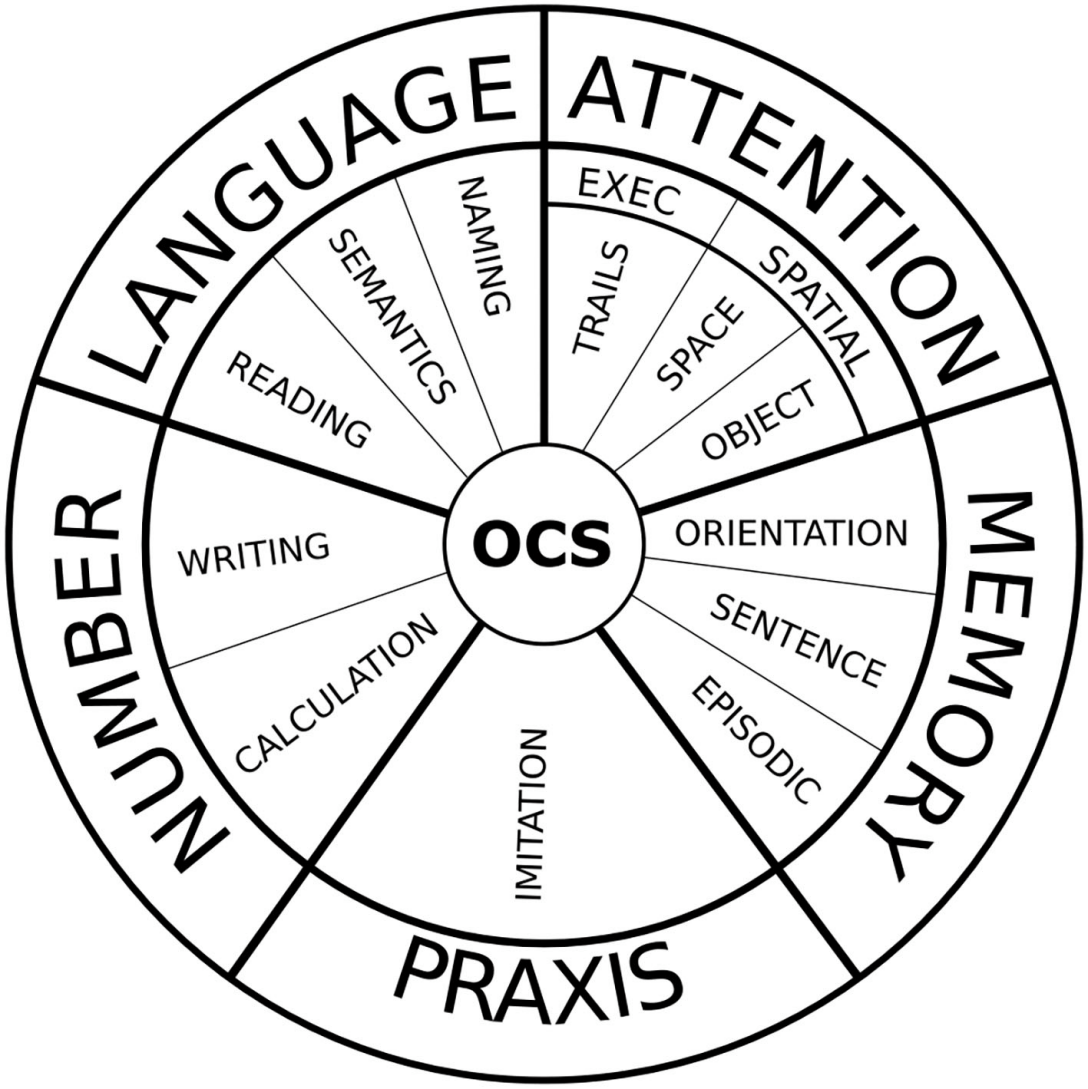
The visual snapshot of the OCS is a compact modality of OCS scoring, in which compromised domains are colored. It provides a quick but informative overview of the cognitive profile of the patient.

The original study classified the OCS subtests under five different theoretical domains: attention (divided into the subdomains of executive functions and visual attention), memory, language, praxis, and number (Figure 1) (4).

A Chinese study tested the reliability of OCS with 5 domains, but the first one was named as “attention and executive function,” and the others were language, memory, number processing, and praxis. The authors found a nearly acceptable level of data-to-model fit, with an improvement in the fitting model obtained when the two subtests related to numerical cognition and praxis were dropped from the model. This yielded an acceptable fit in a model including only three domains:(1) attention and executive function; (2) memory; and (3) language (9). The internal consistency of each of these three domains was tested using Cronbach's alpha coefficient, finding values of 0.3, 0.52, and 0.44 for attention, memory, and language, respectively. These values were lower than the Cronbach's alpha equal to 0.907 evaluated for assessing internal consistency among all the items in a Spanish study (7). This difference could be due to the fact that, in the Chinese study, the Cronbach's alpha was computed on each one of the identified three dimensions on the patients' sample, whereas in the Spanish study, it was computed on all the subtests and collapsing patients and healthy elderly. The Chinese study (9) investigated the structural validity of OCS, but it was done by a confirmatory (and not by an exploratory) factor analysis in which the hypothesis of five and three domains was *a priori* formulated and tested in a sample of 100 patients and 120 controls. Given the known heterogeneity in the cognitive consequences of stroke, it would be important to also carry out exploratory factorial analyses of the OCS on large samples of patients with stroke and healthy controls. Information on this is still limited in the literature.

A recent study conducted on 237 patients with stroke identified only three main components of cognitive functions impaired 1 week after stroke assessed by OCS and the National Institutes of Health Stroke Scale (13). Authors interpreted their results suggesting that neurological deficits following stroke are correlated in a low-dimensional structure of impairment, related neither to the damage of a specific area nor to a vascular territory, but rather reflecting widespread network impairments caused by focal lesions. The first component resulted linked to language, calculation, memory, praxis, and right-sided neglect and was found to be mainly related to left hemisphere damage. The second component was linked to left visuomotor deficits and spatial neglect and mainly related to damage of right cortico-subcortical regions. The third component was linked to right motor deficits and damage in the left subcortical regions. However, the proposed model explained only 50% of the variance, and it was dominated by left hemisphere impairments, similar to other cognitive assessment tools (5, 6). It would appear that while clinicians highlight a high clinical variability among patients with stroke, psychometric tests reveal a limited set of dimensions accounting for a large proportion of variance in performance of the patient with stroke. This could be due to the fact that the large-scale physiological abnormalities following a stroke reduce the variety of neural states visited during task processing and at rest, resulting in a limited repertoire of behavioral states (14).

Overall, a large variability of results and related interpretations emerges from the previous studies on OCS. Presumably, this is due to methodological differences such as whether healthy subjects have been included into the analyses with patients or not, and whether psychometric properties were measured on the OCS in general or on its specific domains.

Despite the general utility of OCS as a cognitive screening tool, the lack in the scientific literature of an exploratory psychometric analysis of OCS domains has led to some critical issues related to its use in clinical routine. A first issue is that in the original OCS under the umbrella domain of attention, executive functions and visuospatial attention are merged, putting together two conceptually different cognitive functions. Even if attention plays a central role in both these functions, neither executive functions nor visuospatial attention can be used to define the impairment of the attention function. This problem also implies that the original OCS does not allow the spatial inattention to emerge as a possible deficit distinct from the attentive component, despite three scores of original OCS could be used to assess unilateral spatial neglect (cancelation, space symmetry, and object asymmetry). Because of the role played by spatial inattention in affecting neurorehabilitative outcomes in patients with stroke (15–17), it would be fundamental to detect and hence to treat this syndrome in a very early phase of stroke. Another critical issue concerns the separation between the “number” and “language” domains in the original OCS; indeed, more recent literature has shown that number writing and calculation should be considered as associated with the language domain (18, 19), indicating the importance of checking the factorial composition of subtests related to linguistic and number processing. These problems may have contributed to the gap between clinicians claiming a high clinical variability among patients with stroke and scientific psychometric tests revealing a limited set of dimensions accounting for a large proportion of variance in the cognitive functions of patients with stroke.

Therefore, the aim of this study was to carry out a factor analysis on a large number of patients with stroke to identify the main OCS domains to solve some scientific and clinical issues related to this useful and valid screening tool.

## Materials and Methods

### Participants

This project represents a secondary analysis of data collected within the UK and Italy. Overall, the OCS was administered to 1,973 patients and 498 healthy participants. In the UK, the data of patients were study data from the Oxford Cognitive Screen (OCS) screening project (n = 416) (4, 5) and the OCS-Care study (*n* = 873) (20) from 2015 to 2019. In Italy, both already collected patients' data (3) and original data were analyzed (*n* = 684). The UK study protocols were reviewed and approved by the National Research Ethics Committee (UK) (references: 11/WM/0299, 14/LO/0648, and 12/WM/00335), and the Italian study protocols were approved by the regional ethics board (Comitato Etico Regione Toscana-Area Vasta Sud Est prot. n.376CEAVSE del 17 12 2015).

The age of patients ranged between 18 and 98 years (mean: 71.91 ± 3.3 years), with mean schooling years of 10.43 ± .9 and 54.8% men. According to the prevalence of stroke, 80.9% of diagnosed cases were of an ischemic origin, 18.8% of hemorrhagic, and 0.3% of other origins. Side of cerebral stroke was the right hemisphere in 51% of cases, left hemisphere in 44%, and the remaining 5% cases were bilateral (extending past the midline or brainstem). A total of 15 patients had a cerebellar stroke (0.08%). The median time from stroke was 6 days (interquartile range: 16 days). Not all the clinical data or OCS items were recorded for all patients, those with a complete dataset being 1,444 (74%). For each analysis, all the available data were used. The age of healthy participants ranged between 18 and 89 years (mean: 53.51 ± 8.4 years); the education years were 12.24 ± .4; for all subjects, there was a complete dataset. Both these values were significantly different from those of patients (*p* < 0.001), presumably because of inclusion or exclusion criteria. Exclusion criteria for healthy subjects were as follows: the presence of previous or ongoing neurological and/or psychiatric disorders, the presence of cognitive decline (as indicated by a Mini-Mental State Examination score lower than (22), the presence of visual field defect as revealed by clinical examination, the presence of visual impairment uncorrected by glasses (3). Given the purpose of the study, it was important that the responses of the healthy group to the tests were not affected by any cognitive or visual impairment, but the above criteria affected the sampling shifting it toward younger and more schooled people. Participants with age <30 years old were the 3% of the full sample, and those with schooling <3 years only the 0.4%.

### OCS Subtests

The OCS is divided into domains and subdomains assessed with specific subtests (for a complete description refer to (3, 4)). The subtests for the language domain are as follows: picture naming (min–max possible range: 0–4), sentence reading (range: 0–15), and semantics (assessed by a picture pointing task; range: 0–3). The subtests for numerical cognition include a subtest of number writing and calculation with two separate subscores: number writing (range: 0–3) and calculation (range: 0–4). The subtest for praxis is imitation (range: 0–12), a task involving meaningless gestures. The subtests of memory include orientation (range: 0–4) and recall and recognition; in this latter subtest, there are separate subscores for sentence recall (range: 0–4) and episodic memory (range: 0–4). Trails (range: −13 /+ 12) is the subtest for attention related to executive functions. Visuospatial attention is assessed by a visual field test (for assessing hemianopia; range: 0–4) and the broken heart cancelation subtest which provides three different subscores: cancelation (i.e., the total number of complete hearts canceled within the time limit as a measure of selective visual attention; range: 0–50), space asymmetry (the difference between complete hearts canceled in the left and right portions of the page as a measure of egocentric neglect; range: −20/+20), and object asymmetry (the difference between left- and right-broken hearts as a measure of allocentric neglect; range: −50/+50). Most of the subtests are formed by 4 items, but semantics (three items) and the trails (two items). Raw data of space and object asymmetry were corrected considering their absolute values, to avoid directionality effects. No scaling corrections were applied to raw data. The total number of obtained subscores is 14.

### Statistical Analyses

The OCS subscores were examined in terms of means and standard deviations according to the previous studies. Data of patients and healthy participants were compared by Mann–Whitney U-test; then, the data of patients with stroke in the left hemisphere were compared with those of the right hemisphere. The alpha level of significance was set at 5%, but it was reduced for multiple comparisons applying Bonferroni correction. A heatmap correlation matrix was computed among all the subtests of OCS using the Pearson correlation coefficient and also partial correlation corrected for demographical factors (age and education). Factor analysis was conducted to identify the main domain in which OCS item scores resulted aggregated by means of principal component analysis (PCA). Being the factors potentially correlated with each other and not totally independent, an obliquity rotation method (direct oblimin method with delta = 0 with Kaiser normalization) was preferred to an orthogonal one. However, because varimax rotation method was often associated with an orthogonal solution often more easily interpretable, we performed a secondary analysis using varimax rotation.

Principal component analysis was conducted on the sample of patients using 14 OCS scores (using absolute values for the space and object asymmetry tasks instead of raw scores to capture both left-sided and right-sided neglect). The selection of the components was performed according to the following criteria suggested by Schonrock-Adema et al. (21): (1) the point of inflection displayed by the scree plot (determined as the maximum or minimum of the derivate of the curve); (2) eigenvalues >1; or (3) eigenvalues with an additional variance of at least 5%. Based on this approach, the choice among the above criteria also depends upon the following criteria about interpretability: (4a) each component should contain variables with a loading ≥ 0.4; (4b) variables loading on the same component should share the same conceptual meaning; (4c) variables loading on different components should appear to measure different constructs; and (4d) most variables should load relatively high on only one component and low on the others. The reliability of PCA results was assessed performing a bootstrapped split-half reliability analysis: patients' data were randomly split into a subsample of 986 individuals, on which a new PCA was conducted; then, a new random split was performed and analyzed. The reliability was assessed computing the Pearson's correlation coefficient (R) on the subtest loadings on corresponding components between the two PCAs and computing the 95% confidence intervals of subtest loadings with respect to their main components.

Being the OCS a screening tool developed for identifying the presence of cognitive deficits in patients with stroke with respect to healthy subjects (more than assessing the level of severity of these deficits within patients' population), we also performed a secondary PCA collapsing data of patients and healthy subjects into a single group for increasing the data variability. At the same time, one may note that this data merging might affect the covariance structure of data, introducing unmatched covariates, and reducing the robustness of the results of PCA calling for caution in its interpretation. These results are reported in the Supplementary Materials.

## Results

### Comparison of Patients With Healthy Subjects

The comparison of scores between healthy participants and patients with stroke confirmed statistically significant differences for all OCS subscores with patients showing higher absolute scores for space and object asymmetry tasks and significantly lower scores in all the other tasks (Table 1). Significant differences were also found among patients with respect to the side of stroke (left hemisphere, right hemisphere, or bilateral, Table 2). The heatmap correlation matrix among the OCS scores showed higher correlations (i) of picture naming with sentence reading, number writing, episodic memory, (ii) of sentence reading with number writing and calculation, (iii) of number writing with calculation, and (iv) of cancelation with imitation, visual field, and space asymmetry (Table 3). The overall Cronbach's alpha (obtained reversing the scores of trails and absolute values of space and object asymmetry) for internal consistency was 0.615. Similar results were found also when correlations were corrected for age and education (Table 3). In general, all the correlations between age or schooling and the patients' scores of OCS subtests had an R <0.25 (the average absolute value of R was 0.10 and 0.15 for age and schooling, respectively), with the only exception of an R = 0.27 between the sentence recalling score and schooling.

**Table 1:** Average scores (mean ± standard deviation) for each group and their comparison carried out with the Mann–Whitney U test (better performances are related to higher values for all the tasks, but trails, object and space asymmetry; for these last two tasks absolute values are reported).

**OCS Domains**	**OCS Tasks**	**Patients**	**Healthy subjects**	***p*-value**
Language	Picture naming	2.81 ± 1.28	3.63 ± 0.62	<0.001
	Semantics	2.84 ± 0.54	3.00 ± 0.00	<0.001
	Sentence reading	12.41 ± 4.36	14.85 ± 0.55	<0.001
Number Cognition	Number writing	2.32 ± 1.0	2.97 ± 0.24	<0.001
	Calculation	3.16 ± 1.10	3.78 ± 0.47	<0.001
Memory	Orientation	3.60 ± 0.90	3.98 ± 0.22	<0.001
	Sentence Recall	2.81 ± 1.61	3.41 ± 0.76	<0.001
	Episodic Memory	3.12 ±1.14	3.87 ± 0.42	<0.001
Attention	Trails	1.82 ± 3.54	−0.43 ± 1.81	<0.001
	Visual Field	3.73 ± 0.70	4.00 ± 0.04	<0.001
	Cancelation	34.44 ± 14.79	47.05 ± 4.0	<0.001
	Object Asymmetry	1.39 ± 2.71	0.15 ± 0.62	0.003
	Space Asymmetry	3.61 ± .67	0.99 ± 1.15	<0.001
Praxis	Imitation	9.07 ± .318	11.40 ± 1.16	<0.001

**Table 2:** Average scores (means ± standard deviation) for each subgroup of patients with respect to side of stroke (the significantly worst performance is highlighted in bold). The *p*-values were computed using Mann–Whitney U-test (in bold if <0.016, based on Bonferroni correction on alpha level of significance).

**OCS Domains**	**OCS Tasks**	**Stroke in left hemisphere**	**Stroke in right hemisphere**	**Bilateral Stroke**	**L vs R**	**L vs B**	**R vs B**	**Cerebellar Stroke**
Language	Picture naming	**2.50 ± 1.43**	2.91 ± 1.15	2.93 ± 1.29	**<0.001**	**0.015**	0.562	2.67 ± 1.29
	Semantics	**2.76 ± 0.66**	2.86 ± 0.51	2.93 ± 0.31	**0.004**	0.048	0.316	3.00 ± 0.00
	Sentence reading	**11.32 ± 5.19**	12.81 ± 3.88	12.57 ± 4.0	**<0.001**	0.211	0.190	13.07 ± 3.71
Number cognition	Number writing	**2.11 ± 1.16**	2.42 ± 0.90	2.27 ± 1.03	**<0.001**	0.434	0.244	2.47 ± 0.99
	Calculation	**2.95 ± 1.25**	3.27 ± 0.99	2.97 ± 1.23	**<0.001**	0.873	0.066	3.47 ± 0.64
Memory	Orientation	3.56 ± 0.91	3.60 ± 0.95	3.41 ± 1.04	0.531	0.407	0.271	3.87 ± 0.35
	Sentence Recall	**2.29 ± 164**	3.01 ± 1.54	2.82 ± 1.40	**<0.001**	**0.003**	0.565	2.67 ± 1.34
	Episodic Memory	**2.91 ± 1.22**	3.22 ± 1.09	3.07 ± 1.20	**<0.001**	0.213	0.324	3.20 ± 0.86
Attention	Trails	**1.49 ± 3.53**	2.32 ± 3.56	2.14 ± 3.87	**<0.001**	0.329	0.434	2.36 ± 4.18
	Visual Field	3.77 ± 0.69	**3.65 ± 0.75**	3.68 ± 0.78	**<0.001**	0.154	0.697	3.71 ± 0.61
	Cancelation	36.82 ± 13.51	**31.04 ± 15.55**	33.10 ± 15.47	**<0.001**	0.117	0.255	36.60 ± 15.73
	Object Asymmetry	−0.29 ± 2.13	**1.18 ± 3.66**	−0.30 ± 2.45	**<0.001**	0.854	**<0.001**	0.87 ± 7.04
	Space Asymmetry	−1.24 ± 4.89	**2.62 ± 6.08**	0.63 ± 5.38	**<0.001**	**0.006**	0.026	2.47 ± 4.55
Praxis	Imitation	**8.58 ± 3.40**	9.21 ± 3.06	9.12 ± 2.62	**0.001**	0.589	0.324	9.21 ± 2.42

**Table 3:** Heatmap correlation matrix for the OCS scores (Pic Nam, picture naming; Sem, semantics; Read, reading; Num. Wr., number writing; Calc, calculation; Ori, orientation; SR, sentence recall; EM, episodic memory; IM, imitation; VF, visual field; Canc., cancelation; O AS, object asymmetry; S AS, space asymmetry; TR, trails).

	**Language**			**Number Cognition**		**Memory**			**Pra-xis**	**Attention**				
	**Pic Nam**	**Sem**	**Read**	**NumWr**	**Calc**	**Ori**	**SR**	**EM**	**IM**	**VF**	**Canc**	**O As**	**S As**	**TR**
Pic Nam	1	0.30	0.43	0.37	0.29	0.23	0.36	0.35	0.33	0.19	0.28	−0.05	−0.09	−0.10
Sem	0.28	1	0.35	0.28	0.27	0.17	0.16	0.24	0.28	0.26	0.23	0.01	−0.08	−0.05
Read	0.45	0.32	1	0.47	0.41	0.25	0.33	0.31	0.26	0.28	0.29	−0.02	−0.08	−0.08
NumWr	0.42	0.26	0.50	1	0.42	0.27	0.26	0.29	0.30	0.24	0.31	−0.08	−0.13	−0.14
Calc	0.36	0.24	0.43	0.47	1	0.32	0.28	0.25	0.25	0.17	0.29	−0.07	−0.14	−0.14
Ori	0.27	0.18	0.24	0.31	0.33	1	0.29	0.28	0.25	0.20	0.29	−0.12	−0.15	−0.09
SR	0.38	0.15	0.31	0.30	0.32	0.29	1	0.40	0.18	0.06	0.12	0.01	−0.02	−0.11
EM	0.41	0.25	0.33	0.36	0.31	0.31	0.42	1	0.25	0.14	0.24	−0.04	−0.03	−0.05
IM	0.39	0.27	0.29	0.37	0.30	0.26	0.22	0.32	1	0.27	0.38	−0.10	−0.16	−0.14
VF	0.20	0.25	0.30	0.24	0.18	0.20	0.08	0.17	0.28	1	0.40	−0.14	−0.22	−0.03
Canc	0.38	0.23	0.33	0.39	0.37	0.32	0.19	0.34	0.44	0.40	1	−0.21	−0.42	−0.20
O As.	−0.12	−0.01	−0.08	−0.16	−0.12	−0.12	−0.02	−0.11	−0.16	−0.16	−0.29	1	0.25	0.16
S As.	−0.17	−0.11	−0.15	−0.19	−0.21	−0.17	−0.07	−0.13	−0.22	−0.25	−0.49	0.31	1	0.09
Trails	−0.21	−0.06	−0.15	−0.23	−0.24	−0.15	−0.19	−0.17	−0.24	−0.08	−0.31	0.22	0.18	1

*Above the diagonal, partial correlations corrected for demographical factors (age and education), below the diagonal, not corrected correlations*.

### Principal Component Analysis

**Figure 2:**
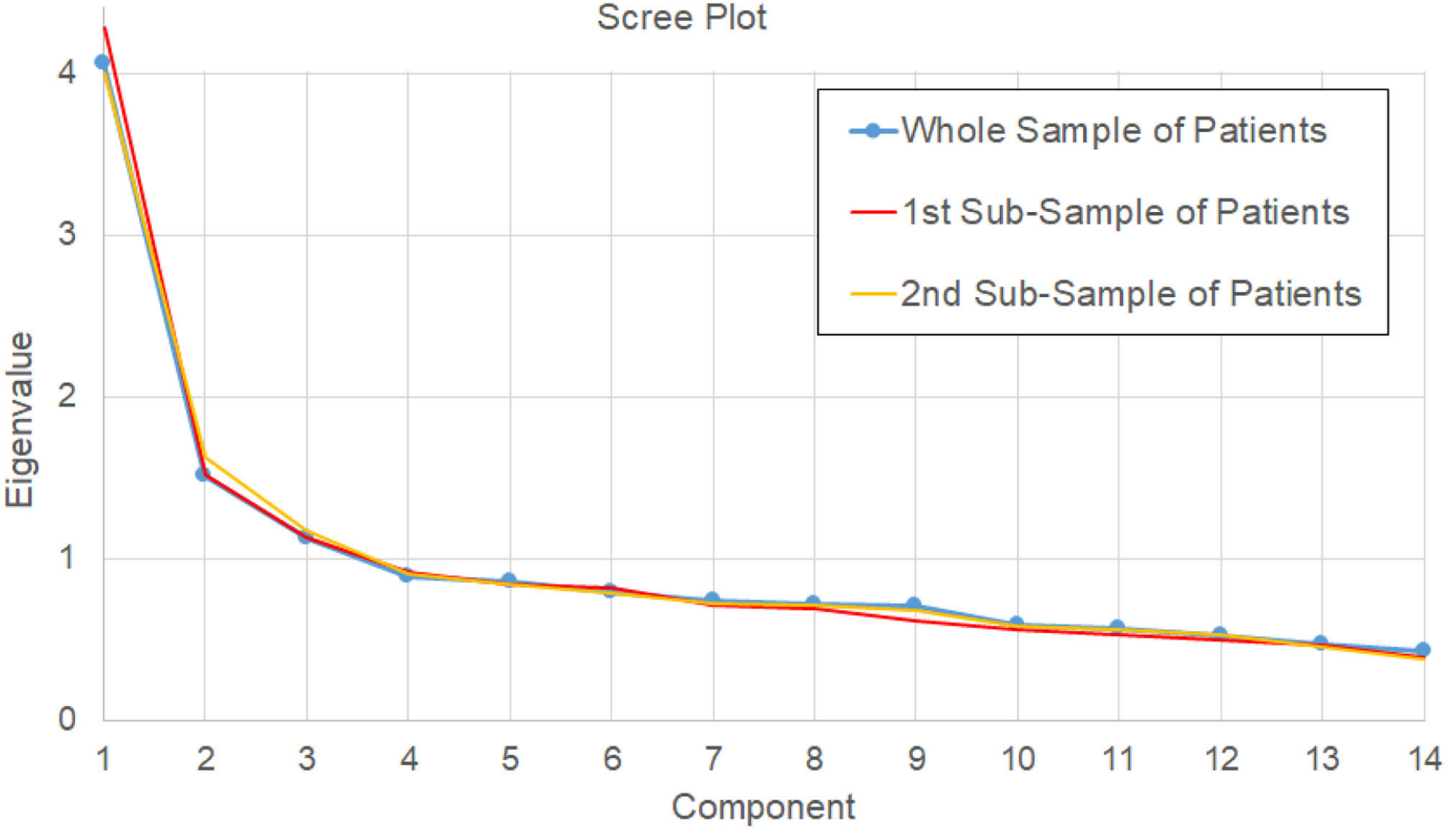
Scree plot of eigenvalues determined by PCA on the 14 scores for the whole sample of patients (main analysis, blue line), and for the two bootstrapped samples of the reliability analysis (red and orange lines).

Performing the PCA on the patients' sample, the scree plot of Figure 2 was obtained. The components with an eigenvalue >1 were three, but they seemed quite different from the three proposed by the three-component proposed model of OCS (9) that were language, memory, attention, and executive functions. Our PCA identified a 1st component that seemed to put together language and memory, being formed by picture naming (0.685), sentence reading (0.612), number writing (0.642), calculation (0.624), imitation (0.404), sentence recall (0.766), episodic memory (0.680), and orientation (0.500). The 2nd component was formed by cancelation (-0.623), object asymmetry (0.691), and space asymmetry (0.734) and seemed related to the unilateral spatial neglect. The 3rd component was formed by semantics (0.553), visual field (0.620), and trails (0.512), with an unclear clinical meaning. The trails subtest also loaded 0.395 on the 2nd component. This model explained less than 50% of variance (48%), keeping out six components with a variance >5% (three with a variance >5.5%).

Analyzing the scree plot a first inflection point was found at the 5th component (a local minimum into the derivate of the scree plot), and a second one at the 6th component (a local maximum). The model with five components maintained the second component related to unilateral spatial neglect as the model with three components, formed by: cancelation (−0.568), object asymmetry (0.678), space asymmetry (0.758). The first component of the three-component model was mainly divided into two components in this new model: one formed by episodic memory (0.757), sentence recall (0.719), and orientation (0.649) and another formed by sentence reading (0.798), number writing (0.732), and calculation (0.766). Semantics (0.670), imitation (0.646), and visual field (0.532) formed another component. The last component was formed by the trails subtest only (0.875). This model explained 60.5% of variance, keeping out only one component with a variance >5.5% (5.6%); however, it violated the criteria 4a and 4d, because picture naming did not achieve the threshold of a loading >0.4, and its loadings were divided between the component related to language (0.306) and that related to memory (0.351), with a low communality (0.511).

The model with six components differed from that with five only because orientation formed a single component, as shown in Table 4, but allowed including all the subtests with an additional variance >5.5%, with each component containing variables with a loading ≥0.4 only on one component. In fact, with respect to the previous model, here, picture naming had a loading >0.4 (0.514) only in the component also formed by sentence recall and episodic memory but not on any other one. The explained variance by this six-component model was 66.1%. All the other eigenvalues showed a variance lower than 5.5%. Table 4 shows the pattern matrix obtained with the PCA for the identified six components. A total of two of these components were mainly formed by a single task: orientation and executive functions (trails).

**Table 4:** The pattern matrix from the principal component analysis on the patients' sample (in bold the higher value for each task, forming clear aggregation of subtasks with absolute values > 0.4).

**OCS Subtask**	**Components**						**Communality**	**95% CI main load**
	**1**	**2**	**3**	**4**	**5**	**6**		
Sentence Reading	**0.771**	0.006	0.123	0.093	0.128	−0.159	0.699	0.66–0.76
Number Writing	**0.713**	−0.051	−0.083	0.074	0.032	0.010	0.611	0.64–0.78
Calculation	**0.761**	0.013	−0.129	−0.055	−0.102	0.250	0.678	0.78-0.85
Cancelation	0.115	–**0.430**	−0.166	0.019	0.383	0.241	0.642	0.34-0.64
Object Asymmetry	0.004	**0.852**	0.055	−0.132	0.178	0.211	0.723	0.46–1.00
Space Asymmetry	−0.024	**0.676**	−0.021	0.101	−0.121	−0.201	0.592	0.60–0.96
Trails	−0.083	0.053	**0.921**	0.082	0.000	0.035	0.860	0.91–0.91
Sentence Recall	0.137	0.060	−0.043	**0.721**	−0.161	0.148	0.640	0.65–0.86
Episodic Memory	−0.061	−0.080	0.088	**0.808**	0.111	0.090	0.681	0.80–0.82
Picture naming	0.278	−0.077	−0.056	**0.514**	0.214	−0.155	0.590	0.31–0.73
Semantics	0.175	0.166	0.048	0.078	**0.666**	−0.135	0.556	0.63–0.74
Visual Field	0.107	−0.228	0.202	−0.151	**0.609**	0.149	0.581	0.49–0.70
Imitation	−0.110	0.016	−0.342	0.214	**0.629**	0.076	0.615	0.54–0.68
Orientation	0.092	0.035	0.024	0.230	−0.006	**0.813**	0.792	0.69–0.93

*The last two columns report the results of the communality table on the whole sample of patients and the 95% confidence interval of the main load for each subtest obtained by the reliability analysis of the two subsamples of patients*.

### Reliability Analysis

**Figure 3:**
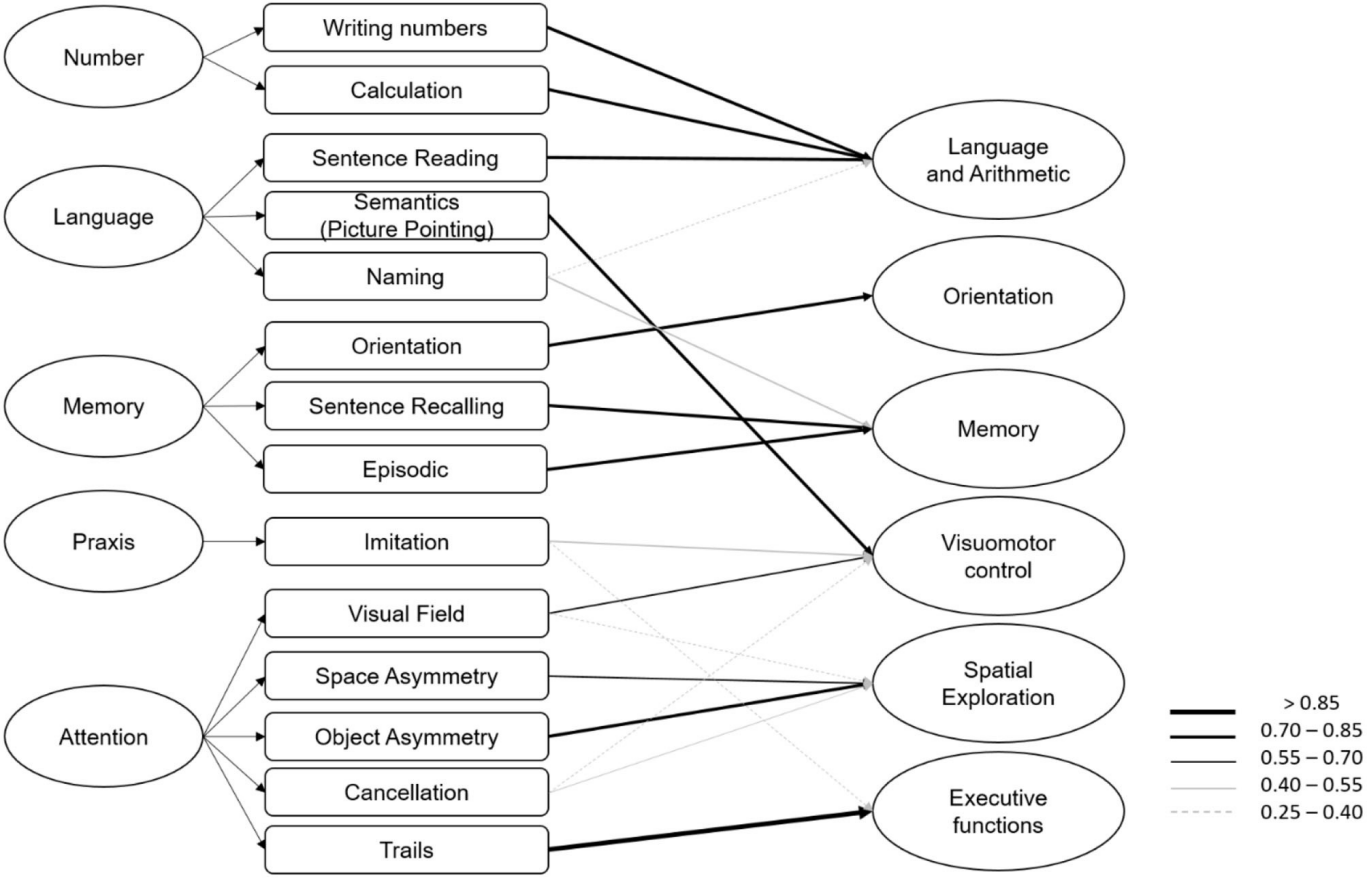
On the left the original structure of OCS with five domains and on the right the six components identified by the principal component analysis, with arrows reported for values >0.25 according to the legend.

We tested the bootstrapped split-half reliability by randomly splitting the data of patients into two subgroups, running the PCA, and comparing the results obtained for the two subsamples. We obtained results similar to those obtained for the entire sample. The scree plots of these two analyses are reported in Figure 3, with the first inflection point observed at the 5th component for one PCA, and at the 6th component for the other one. Similar to the main PCA performed on the whole sample of patient, these two PCAs explained the 67 and 68% of variance, respectively. The six-component model satisfied the above-reported criteria in both cases (21). The correlations between the loadings of the subtests in the two subgroups were all statistically significant. Referring to the order of components reported in Table 4, the absolute values of R were highly significant (*p* < 0.001); for four components, R was >0.9 (*p* < 0.001), for the 4th component (related to memory) R = 0.86 (*p* < 0.001) and for the 6th component (orientation) R = 0.66 (*p* = 0.011). This reliability analysis allowed also identifying the 95% confidence intervals of the loadings of each subtest with respect to its main component. Only the cancelation and picture naming subtests had an interval crossing the threshold of 0.4 (criterion 4a and 4d), despite achieving in both the subsamples a main loading >0.4.

Then, we performed a PCA on all the data combining patients' and healthy subjects' data: the results did not change with six components overcoming the cutoff, formed by the same tasks identified by the main analysis (details are reported in the Supplementary Materials). The explained variance of the PCA performed on patients and healthy subjects was slightly increased (67.5 vs. 66.1%), and the number of required rotations was reduced (9 vs. 19). Finally, we repeated this last analysis changing the rotation method using the varimax rotation instead of obliquity rotation and results did not change (for details refer to Supplementary Materials).

## Discussion

Our results showed alterations in all the OCS subtests in patients with stroke with respect to healthy subjects also with high levels of statistical significance, confirming the sensitivity of the tasks into detecting cognitive alterations (5). Also, specificity was confirmed by the significant differences found between patients with left versus right stroke (20).

The PCA identified components quite different from those originally proposed. Hong et al. (9) already proposed a revision of the OCS with three main domains, but simply removing the domain of number and that of praxis. It is important to note that their study was conducted only on patients without unilateral spatial neglect. Indeed, they highlighted the need of studies reviewing the existing five-dimensional domains for improving the structural validity and internal consistency of OCS also for patients with neglect. In our study, which includes also patients with unilateral spatial neglect, one of the domains, independently by the chosen number of components (three, five, or six), was formed by cancelation, object asymmetry, and space asymmetry, which is conceivably related to unilateral spatial neglect. In the original version of OCS, these subtests were associated also with the trails subtest and referred to the domain of attention (subdivided into spatial attention and executive functions). Also in our study, a model based on three components showed the additional loading of the trails subtest in this last domain. However, this model explained only 50% of variance, had a large component including eight subtasks, and excluded three components with an additional variance higher than 5.5%. The models with 5 and 6 components differed from each other only for the orientation subtest that, in the former, was aggregated to the memory domain as in conventional OCS, while, in the latter model, was defined as an independent component. With respect to the five-component model, that with six components had three advantages: (a) it included all the subtests with an additional variance >5.5% (criterion three of (21)); (b) each component contained variables with a loading ≥0.4 (criterion 4d of (21)); and (c) variables loaded relatively high on only one component and low on the others (criterion 4d of (21)).

The bootstrap split-half analysis confirmed the reliability of the model with six components, with statistically significant correlations between the results of the two PCAs performed on patients' subsamples. Furthermore, the 95% confidence intervals showed high values of loadings for each subtest only on one of the six components, in keeping with the above-defined criteria for interpreting PCA results (21). The robustness of our results was also confirmed by the fact that they did not change by varying the rotation method of the PCA. Finally, when patients' data were analyzed together with those of healthy subjects, the PCA identified the same six components of the main analysis (as detailed in the Supplementary Materials).

### Differences With Original OCS Structure

Independently of the number of components, our study also highlighted some differences with respect to the original classification, and, in particular, the existence of a domain related to unilateral spatial neglect, the aggregation of arithmetic subtaskswith that of sentence reading subtest, and the unexpected aggregation of semantics subtest with the visual field subtest. First of all, our PCA identified a first component mainly formed by the sentence reading, number writing, and calculation subtests. Associations between some aspects of reading and arithmetic, two cognitive skills learned during schooling, have long been supported by behavioral, brain lesion, and functional brain imaging studies (18, 19). The relationships that exist between some specific aspects of arithmetic and left hemisphere language were also reported by cognitive development research. These studies have showed that children's reading and mathematics activity converged in prefrontal cortex across multiple tasks, but dissociated in temporal and parietal cortices, showing similarities to the adult pattern of dissociation (18). As posited by the “triple-code model” of number processing (22–24), of the three systems of representations of numerical information (quantitative, verbal, and visual), the quantitative system is unique to numerical processing, whereas the verbal and visual systems share aspects with language processing. We note here that picture naming and semantics did not directly contribute to this component. This first latent component, therefore, was considered to relate to “Language and arithmetic.”

In our study, the semantics subtest was found to be mainly involved in another component together with visual field (already in the 3-component model) and praxis (in the models with five and 6 components). Though this may seem surprising, it should be noted that the semantics subtest in the OCS is assessed by asking the patient to point with the hand to the drawing representing a word read by the researcher. This means that the task in essence is a picture pointing task. Some evidence suggests an interaction between the ventral visual-perceptual and the dorsal visuomotor brain systems during the course of object recognition (25). In the praxis task, the patient is required to mimic the gestures performed by the researcher. Furthermore, in the visual field subtest, the patient is asked to look at the examiner's nose and point to the moving hand. Since all these tasks could be hence related to visual attention and motor responses, this third component can be considered as related to “Visuomotor control.”

The domain of memory was quite preserved in our models, with a component including sentence recall and episodic memory, but also the picture naming subtest (that in the original OCS was associated with language domain). The differences between our five- and six-component models are mainly related to this domain. In the five-component model, the picture naming subtest had a low loading (0.351, <0.4) and this component also included the orientation subtask (loading: 0.649). In the 6-component model, the picture naming subtest had a high loading (0.514) whereas the orientation subtest formed a single sub-test component (loading: 0.813). Hence, picture naming resulted related to semantic memory. As highlighted by a recent study, not all putative tests of semantic and episodic memory may necessarily measure the hypothesized construct, and there is a conceivable overlapping between these cognitive functions (26). The orientation subtask could be associated with memory domain or resulting in a separate domain factor instead of forming a part of a wider memory classification. For basic orientation to time and place to be impaired, patients usually present a severe cognitive impairment (even delirium or related to pre-existing dementia). Similarly, other cognitive scales consider orientation as a stand-alone cognitive domain, such as the Mini-Mental State Examination (based on five different domains: orientation, working memory, memory recall, language, visuospatial motor functions and a fifth domain related to attention, concentration, and calculation) and the Montreal Cognitive Assessment (based on ten domains) (27).

The main difference in our analyses with respect to the domains of the original version of OCS was the presence of a component clearly related to the presence of “Unilateral spatial neglect,” being formed by the cancelation, object asymmetry, and space asymmetry measures of the broken heart subtest. This component is therefore referred to as “Spatial exploration function.” As shown by the comparison of patients with left and right stroke, the latter ones had a more severe neglect, whereas the former had a score with the opposite sign. Visual field partially contributed to this component, but mainly to the visuomotor control domain. On the other hand, cancelation had a slight contribution also to visuomotor control domain, probably in terms of visuomotor attention. The presence of unilateral spatial neglect also reduces the motor skills re-acquired by patients with stroke during neurorehabilitation (15). It should be noted that in the OCS, peripersonal, but not personal, neglect is considered, and these two deficits may recover independently (28).

Finally, the trails subtest marked another component which may be interpreted as related to “Executive functions,” a domain considered as independent also in the Montreal Cognitive Assessment (27).

Importantly, neither the model with three components nor that with six components defined attention as a separate domain. This could be due to different intertwined reasons. Attention can be seen as a control function with a cross-test influence. At the same time, many different types of attention exist (selective attention, divided attention, sustained attention, and so on) and their impairment could lead to different cognitive deficits, and in turn, they can influence the performance of other cognitive functions.

Our results could be summarized as follows. Our PCA showed that some differences in how sub-tests should be aggregated into domains, with respect to the original version of OCS. The model with three principal components matched the criterion of eigenvalues >1, which was in line with previous results (9, 13), had a clear meaning, but it could be only poorly useful because too simple (explaining only the 50% of variance). The model with five components matched the criterion of the first inflection point to determine the number of components, but the picture naming subtest had loadings on two components instead of only one. The model with 6 components matched the criterion of including components with an additional variance higher than 5.5%, and it was associated with a second inflection point. With respect to the five-component model, this one just associated a specific component to the orientation subtest, and it solved the problem of loadings on more components, facilitating the interpretability of results (criterion 4d).

### Implications of the Present Analyses for the Clinical Use of OCS

Our results highlighted some important warnings that could be helpful for clinicians using the OCS. First of all, the domain called “Number” was found to be only related to that of language. Then, the subtest called semantics and hence referred to language includes a picture pointing subtask and may be related to visuomotor deficits even more than language deficits. Similarly, praxis is evaluated using the imitation subtest that requires visuomotor abilities. The picture naming subtest also includes the involvement of memory function in terms of semantic memory and loaded on this domain. Orientation proved very important and was found as a separate factor, suggested to be related to severe cognitive impairment. Finally, attention was already divided in the original OCS partly into executive functions and partly into visuospatial attention: the measures of cancelation task, space, and object asymmetry of the broken heart sub-test were clearly related to spatial exploration and hence to the possible presence of unilateral spatial neglect, whereas the visual field subtest was more related to visuomotor control.

**Figure 4:**
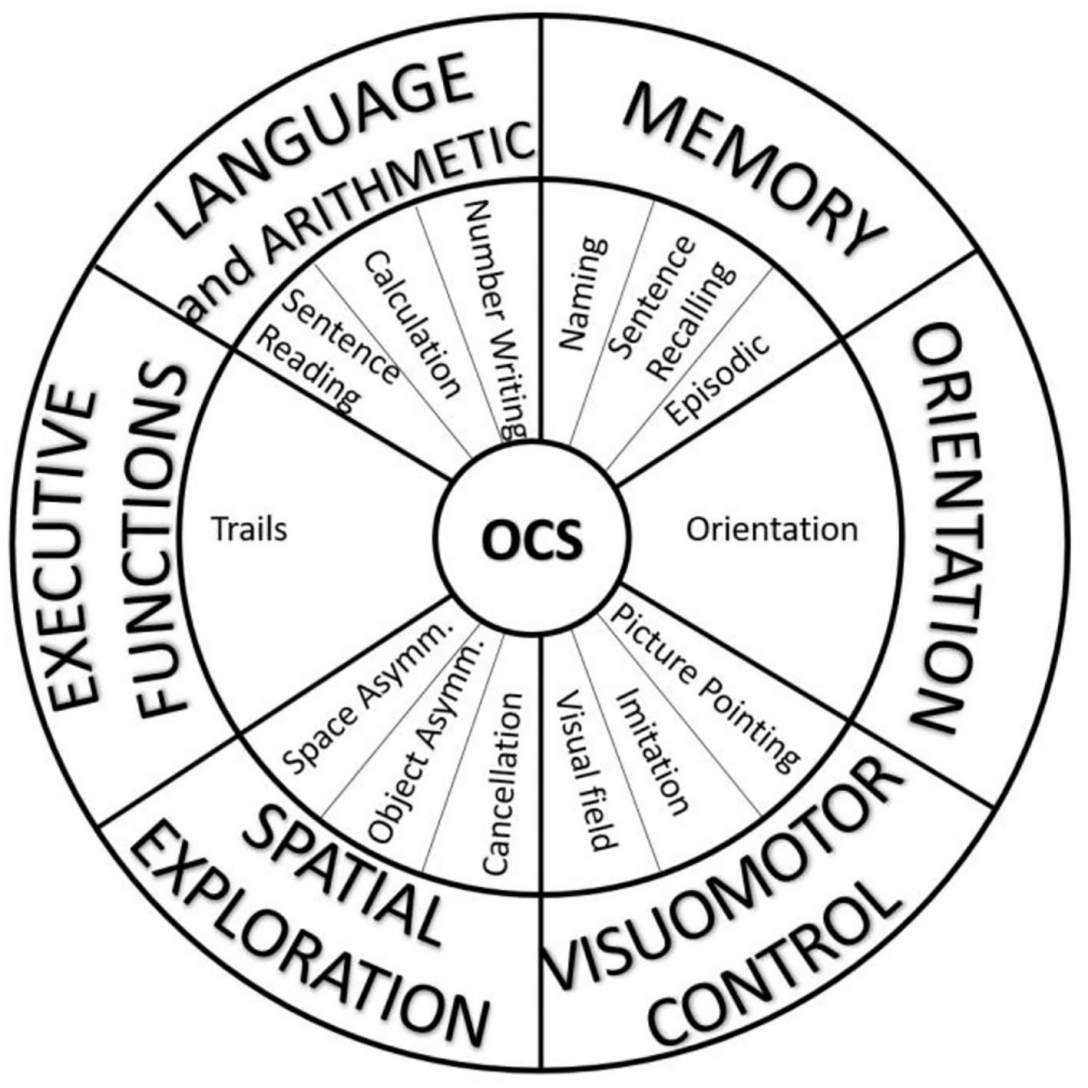
The alternative visual snapshot of OCS developed in accordance with the results of principal component analysis. The subtasks (middle ring) remain the same, but the domains (external ring) are different from the original version.

Though the theoretical model of OCS can mainly be considered preserved, a more complex distribution of the weights of each subtest into different domains emerged from our analyses. Clinicians could effectively continue to use the OCS for the early assessment of cognitive deficits in patients with stroke, adopting the classical version of the visual snapshot. However, we propose here a slightly different version with the aim to take into account the results of our analyses. This new snapshot of OCS maintains the same subtests, subscores, and materials (test booklet and patient pack) but is redefined based on the alternative approach related to the six domains found in the present PCA (Figure 4): language and arithmetic, memory, visuomotor control, orientation, spatial exploration, and executive functions. In clinical practice, the new snapshot may be more useful for rehabilitation treatment compared to the original one, as it allows the team immediately focusing on the impaired cognitive domain such as attention (cancelation results in selective attention), spatial orientation (egocentric versus allocentric neglect), and executive functions. The impairment of these cognitive abilities plays a central role in rehabilitative recovery.

### Study Limitations

The findings of our study should be considered in the light of some limitations. The main limit is that being focused on a principal component analysis of OCS, we did not use other cognitive assessment scales. Another limit of our study is the absence of information about lesion size that is an important factor related to stroke severity. However, previous studies already compared OCS scores with other cognitive assessment tools, proving the validity and reliability of OCS. Furthermore, we did not correct the data for the age or schooling of participants to simplify an already complex analysis (these corrections were limited to the correlation heatmap). This choice was due to two main reasons: (1) previous results showed that demographic variables had quantitatively small effects on the scores of OCS tasks (3),(2) a previous study showed that these effects could be modeled with different equations among tasks (3), (3) we were more interested in within-subject clusterization of items into domains than in between-subject comparisons.

It is important to note that the healthy group enrolled in our study was significantly younger and more schooled than the patients. This could be considered as a sampling bias of our study, related to the difficulties of finding subjects without any neurological or visual deficits over 70 years old (that was the mean age of patients). On one hand, according to the aim of this study, it was more important that the answers of healthy group were not affected by any deficits than matching age and schooling, as done in the original study about OCS, in which the same sampling bias was already present (4). On the other hand, the literature lacks a matched case–control study conducted resampling the groups by pairing age and schooling, despite it will probably reduce the width of samples. Then, the cognitive functions of patients widely vary among acute, subacute, and chronic phases of stroke. In our study, the median time from the acute event and the cognitive screening was 6 days (with an interquartile range of 15 days); so, our sample is mainly the representative of acute and subacute population, when the OCS is mainly used to define a personalized rehabilitation program.

So, the OCS is a helpful screening tool for cognitive functions, but its meaning and utility may depend on its interpretation that is left to the clinicians and it may depend on the stroke phase in which the patient is, especially in some domains. In particular, the assessment of orientation could be fundamental in the acute phase and less in the chronic one. On the other hand, an orientation deficit could also be detected in the chronic phase, being clinically relevant because attributable to different specific processes (e.g., degenerative processes). The results of our study could be helpful for helping clinicians in this interpretation because improved the definition of the cognitive domains covered by OCS subtests.

## Conclusion

Overall, the Oxford Cognitive Screen has already been validated as a useful tool for an easy and early screening of cognitive deficits in patients with stroke (4, 5). With the analyses reported in our study, we provided important further information about the meaning of the OCS subtests and their weights on specific cognitive domains. Even though the subtests of OCS are relatively simple, and each aims to measure a particular domain, nevertheless, a wider set of functions is involved in their execution. This pertains most clearly to the required motor responses and visuomotor coordination in some of the tasks. Based on these analyses, we proposed a new visual snapshot expressing the OCS subtests as a function of the six domains found: language and arithmetic, memory, visuomotor control, orientation, spatial exploration, and executive functions. We hope that this further information and caution about the OCS domains and/or the refinement of a new snapshot for the OCS may favor its clinical use by improving the tuning in the description of the patient's cognitive impairments.

## Data Availability Statement

The raw data supporting the conclusions of this article will be made available by the authors, without undue reservation.

## Ethics Statement

The studies involving human participants were reviewed and approved by National Research Ethics Committee (UK). The patients/participants provided their written informed consent to participate in this study.

## Author Contributions

MM, and ND conceptualized the study. LA supervised the data collection. MI analyzed the data and wrote the first draft of this manuscript. MM, ND, and PZ supervised the study and provided important contributions to the draft. All authors contributed to the article and approved the submitted version.

## Funding

This work was supported by the awards from the Tuscany Rehabilitation Clinic, Montevarchi, Arezzo, Italy Stroke Association (TSA 2011/02 and TSA LECT 2015/02), by the National Institute for Health Research (NIHR) Oxford Biomedical Research Center (BRC), by the Project of Excellence Psychological Adaptation to ever Changing Environments obtained by the Department of Psychology of Sapienza University.

## Conflict of Interest

The authors declare that the research was conducted in the absence of any commercial or financial relationships that could be construed as a potential conflict of interest.

## Publisher's Note

All claims expressed in this article are solely those of the authors and do not necessarily represent those of their affiliated organizations, or those of the publisher, the editors and the reviewers. Any product that may be evaluated in this article, or claim that may be made by its manufacturer, is not guaranteed or endorsed by the publisher.
