# Supplementary material for: Principal Component Analysis of Oxford Cognitive Screen in Patients With Stroke
Source: Front Neurol. 2022 May 27;13:779679. doi: 10.3389/fneur.2022.779679 (PMC9197217; doi:10.3389/fneur.2022.779679)
Supplement: Supplementary file 1 [file Table_1.DOCX]

Principal component analysis of Oxford Cognitive Screen in patients with stroke

Marco Iosa^1,2^, Nele Demeyere^3^, Laura Abbruzzese^4^, Pierluigi Zoccolotti^1,2^, Mauro Mancuso^5,4*^

^1^Department of Psychology, Sapienza University of Rome, Rome, Italy

^2^ IRCCS Fondazione Santa Lucia, Rome, Italy

^3^ Department of Experimental Psychology, University of Oxford, Oxford, UK.

^4^ Tuscany Rehabilitation Clinic, Montevarchi, Arezzo, Italy

^5^ Physical and Rehabilitative Medicine Unit, NHS-USL Tuscany South-Est, Grosseto, Italy

**SUPPLEMENTARY MATERIAL**

**Table S1**. *Demographical and clinical data for the patient sample (also divided in the three original samples: Italian Cohort, OCS screening project and OCS care study) and the healthy control group.*

| **Demographical and clinical features** | | **Patient Samples** | | | | **Healthy Control Group** |
| --- | --- | --- | --- | --- | --- | --- |
|  |  | **Italian Cohort** | **OCS screening project** | **OCS-care Study** | **Entire sample of Patients** |  |
| Sample size | | 684 | 416 | 873 | 1973 | 498 |
| Age (years) | | 71.1±12.8 | 73.8±13.5 | 71.6±13.6 | 71.9±13.3 | 53.5±18.4 |
| Education (years) | | 8.6±4.4 | 11.4±2.5 | 11.9±2.9 | 10.4±3.9 | 12.2±4.4 |
| Gender | Female | 48% | 46% | 43% | 44% | 45% |
|  | Male | 52% | 54% | 57% | 56% | 55% |
| Type of stroke | Ischemic | 76.8% | 78.7% | 82.8% | 80.9% | - |
|  | Hemorrhagic | 22.6% | 16.4% | 15.6% | 18.8% |  |
|  | Others | 0.06% | 4.9% | 1.6% | 0.3% |  |
| Side of lesions | Right side | 54.2% | 49.4% | 47.9% | 51% | - |
|  | Left side | 39.5% | 45.9% | 47.0% | 44% |  |
|  | Bilateral | 4.0% | 4.7% | 5.1% | 5% |  |
|  | Cerebellar | 2.3% | 0.0% | 0.0% | 0% |  |

| **OCS Sub-task** | **Components** | | | | | |
| --- | --- | --- | --- | --- | --- | --- |
|  | **1** | **2** | **3** | **4** | **5** | **6** |
| Sentence Reading | **0.776**  **0.751**  **0.771** | 0.108  0.251  0.093 | 0.144  0.275  0.128 | -0.146  -0.056  -0.159 | -0.011  -0.066  0.006 | -0.131  -0.064  0.123 |
| Calculation | **0.766**  **0.731**  **0.761** | -0.071  0.111  -0.055 | -0.102  0.049  -0.102 | 0.233  0.308  0.250 | 0.025  -0.080  0.013 | 0.138  0.196  -0.129 |
| Number Writing | **0.723**  **0.714**  **0.713** | 0.069  0.231  0.074 | 0.026  0.168  0.032 | 0.005  0.094  0.010 | -0.064  -0.144  -0.051 | 0.072  0.136  -0.083 |
| Episodic Memory | -0.057  0.118  -0.061 | **0.790**  **0.765**  **0.808** | 0.111  0.196  0.111 | 0.089  0.147  0.090 | -0.094  -0.120  -0.080 | -0.049  0.019  0.088 |
| Sentence Recall | 0.108  0.230  0.137 | **0.749**  **0.742**  **0.721** | -0.183  -0.074  -0.161 | 0.142  0.202  0.148 | 0.075  0.048  0.060 | 0.020  0.085  -0.043 |
| Picture Naming | 0.263  0.374  0.278 | **0.530**  **0.587**  **0.514** | 0.188  0.282  0.214 | -0.166  -0.080  -0.155 | -0.083  -0.124  -0.077 | 0.072  0.131  -0.056 |
| Semantics | 0.136  0.213  0.175 | 0.082  0.155  0.078 | **0.721**  **0.721**  **0.666** | -0.084  -0.040  -0.135 | 0.205  0.124  0.166 | 0.009  0.013  0.048 |
| Visual Field | 0.105  0.164  0.107 | -0.126  -0.058  -0.151 | **0.608**  **0.630**  **0.609** | 0.167  0.186  0.149 | -0.278  -0.345  -0.228 | -0.179  -0.146  0.202 |
| Imitation | -0.071  0.087  -0.110 | 0.221  0.301  0.214 | **0.528**  **0.551**  **0.629** | 0.056  0.109  0.076 | -0.044  -0.149  0.016 | 0.397  **0.408**  -0.342 |
| Orientation | 0.077  0.171  0.092 | 0.197  0.248  0.230 | -0.002  0.099  -0.006 | **0.850**  **0.857**  **0.813** | 0.032  -0.085  0.035 | -0.013  0.043  0.024 |
| Object Asymmetry | -0.016  -0.061  0.115 | -0.105  -0.115  -0.132 | 0.190  0.125  0.178 | 0.160  0.128  0.211 | **0.836**  **0.798**  **0.852** | -0.036  -0.090  0.055 |
| Space Asymmetry | -0.021  -0.084  -0.024 | 0.068  0.017  0.101 | -0.122  -0.179  -0.121 | -0.152  -0.172  -0.201 | **0.694**  **0.723**  **0.676** | -0.021  -0.070  -0.021 |
| Cancellation | 0.117  0.229  0.115 | 0.048  0.153  0.019 | 0.331  **0.407**  0.383 | 0.205  0.256  0.241 | **-0.450**  **-0.543**  -0.430 | 0.214  0.262  -0.166 |
| Trails | -0.073  -0.147  -0.083 | 0.062  -0.076  0.082 | 0.051  0.010  0.001 | 0.027  -0.030  0.035 | 0.051  0.155  0.053 | **-0.915**  **-0.895**  **0.921** |

***Table S2****. The pattern matrix from the Principal Component Analysis on the entire sample when oblique (black) or varimax (blue) rotation was applied, and on the only sample of patients as reported in the main manuscript (red). The data are in bold if the higher value for each task, forming clear aggregation of sub-tasks with absolute values >* *0.4 as reported in Table 4 of the paper. As shown, the results are very similar in the three conditions, with six components identified independently by the methods or the samples.*
